# Synthesis, Characterization and Solution Chemistry of *trans*-Indazoliumtetrachlorobis(Indazole)Ruthenate(III), a New Anticancer Ruthenium Complex. IR, UV, NMR, HPLC Investigations and Antitumor Activity. Crystal Structures of *trans*-1-Methyl-Indazoliumtetrachlorobis-(1-Methylindazole)Ruthenate(III) and its Hydrolysis Product *trans*-Monoaquatrichlorobis-(1-Methylindazole)-Ruthenate(III)

**DOI:** 10.1155/MBD.1996.243

**Published:** 1996

**Authors:** Kari-Georg Lipponer, Ellen Vogel, Bernhard K. Keppler

**Affiliations:** 1 Institute of Inorganic Chemistry University of Heidelberg Im Neuenheimer Feld 270 Heidelberg D-69120 Germany

## Abstract

Besides intensive studies into the synthesis of the complex *trans*-Hlnd[RuCl_4_(ind)_2_] (Ind = indazole) **1**, which differs remarkably from the usual method for the complexes of the HL[RuCl_4_L_2_] - type, competitive products and hydrolysis of this species are described. Stability and pseudo-first-order rate constant under physiological conditions of complex **1** in comparison with the analogous imidazole complex *trans*-Hlm[RuCl_4_im_2_] (Im = imidaZole) **ICR** were examined by means of HPLC, UV and conductivity measurements (K_obs._(**1**) = 1.55 × 10^-4^ s^-1^; K_obs._(**ICR**) = 9.10 × 10^-4^ s^-1^). An attempt was made to elucidate the bonding conditions in **1** by studying the reactions of Ru(lll) and the two N-methyl isomers of indazole. It can be expected that bonding in the unsubstituted ligand should occur via the N2 nitrogen. The molecular structures of the complex *trans*-H(1-Melnd)[RuCl_4_(1-Melnd)_2_] × 1H_2_O (1-Melnd = 1-methylindazole) **6** and its hydrolysis product in aqueous solution [RuCl_4_(H_2_O)(1-Melnd)_2_] **7** were determined crystallographically. After anisotropic refinement of F values by least squares, *R* is 0.053 for **6** and 0.059 for **7**. Both complexes crystallize with four molecules in a unit cell of monoclinic symmetry. The space group is *P2.1/n* for **6** with cell dimensions *a* = 10.511Å, *b* = 13.87Å, *c* = 19.93Å, and β = 98.17° and *C2/c* for **7** with *a* = 19.90Å, *b* = 10.94Å, *c* = 8.490Å and β = 96.74 ° The fact that the aqua species **7** could be isolated after dissolving **6** in a water/acetone solution confirmed the theory of many Ru(lll) complexes being initially transformed, under physiological conditions, into aqua complexes in a first and often rate-determining hydrolysis step. Compounds **1** and **ICR** are potent antitumor agents which exhibit activity against a variety of tumor cells and experimental tumor models in animals, including autochthonous colorectal tumors. Clinical studies with **1** are in preparation.


### SYNTHESIS, CHARACTERIZATION AND SOLUTION CHEMISTRY OF *trans*-INDAZOLIUMTETRACHLOROBIS(INDAZOLE)RUTHENATE(III), A NEW ANTICANCER RUTHENIUM COMPLEX. IR, UV, NMR, HPLC INVESTIGATIONS AND ANTITUMOR ACTIVITY. CRYSTAL STRUCTURES OF *trans*-1-METHYL-INDAZOLIUMTETRACHLOROBIS-(1-METHYLINDAZOLE)RUTHENATE(III) AND ITS HYDROLYSIS PRODUCT *trans*-MONOAQUATRICHLOROBIS-(1-METHYLINDAZOLE)-RUTHENATE(III)

Karl-Georg Lipponer, Ellen Vogel and Bernhard K. Keppler*

Institute of Inorganic Chemistry, University of Heidelberg, Im Neuenheimer Feld 270, D-69120 Heidelberg, Germany

Besides intensive studies into the synthesis of the complex *trans*-HInd[$RuCl_4(ind)_2$] (Ind = indazole) **1**, which differs remarkably from the usual method for the complexes of the HL[$RuCl_4L_2$] - type, competitive products and hydrolysis of this species are described. Stability and pseudo-first-order rate constant under physiological conditions of complex **1** in comparison with the analogous imidazole complex *trans*-HIm[$RuCl_4(im)_2$] (Im = imidazole) **ICR** were examined by means of HPLC, UV and conductivity measurements ($k_{obs.}$(**1**) = 1.55 x $10^{-4}$ $s^{-1}$; $k_{obs.}$(**ICR**) = 9.10 x $10^{-4}$ $s^{-1}$). An attempt was made to elucidate the bonding conditions in **1** by studying the reactions of Ru(III) and the two N-methyl isomers of indazole. It can be expected that bonding in the unsubstituted ligand should occur via the N2 nitrogen. The molecular structures of the complex *trans*-H(1-MeInd)[$RuCl_4$(1-MeInd)$_2$] x $1H_2O$ (1-MeInd = 1-methylindazole) **6** and its hydrolysis product in aqueous solution [$RuCl_3(H_2O)$(1-MeInd)$_2$] **7** were determined crystallographically. After anisotropic refinement of F values by least squares, *R* is 0.053 for **6** and 0.059 for **7**. Both complexes crystallize with four molecules in a unit cell of monoclinic symmetry. The space group is *P2.1/n* for **6** with cell dimensions $a$ = 10.511Å, $b$ = 13.87Å, $c$ = 19.93Å and $\beta$ = 98.17° and *C2/c* for **7** with $a$ = 19.90Å, $b$ = 10.94Å, $c$ = 8.490Å and $\beta$ = 96.74°. The fact that the aqua species **7** could be isolated after dissolving **6** in a water/acetone solution confirmed the theory of many Ru(III) complexes being initially transformed, under physiological conditions, into aqua complexes in a first and often rate-determining hydrolysis step. Compounds **1** and **ICR** are potent antitumor agents which exhibit activity against a variety of tumor cells and experimental tumor models in animals, including autochthonous colorectal tumors. Clinical studies with **1** are in preparation.

### Introduction

Today the development of ruthenium complexes as an alternative to platiniferous tumor inhibitors is of special interest[1-8]. Ruthenium complexes with the general formula HL[$RuCl_4L_2$], with L = nitrogen heterocycle, show antitumor activity in different systems *in vitro* and *in vivo* and outstanding activity in an autochthonous colorectal tumor model with a tumor reduction of about 70 % to 90%[3,9-14]. The water-soluble anionic complexes are characterized by *trans-* standing heterocycles. The first complex of the type mentioned above we synthesized was *trans*-HIm[$RuCl_4(im)_2$] **(ICR)**, with two imidazoles as organic ligands[9]. Now we present the analogous indazole complex *trans*-HInd[$RuCl_4(ind)_2$] **(1)**, which shows higher antitumor activity than **ICR** in many test systems. The structures of **ICR** and **1** are shown in Figure 1. *trans*-HInd[$RuCl_4(ind)_2$] **(1)** is not only active against transplantable tumors but also in a model system with acetoxymethylmethylnitrosamine (AMMN) - induced tumors in the colon of rats. These autochthonous tumors induced by intrarectal application of this carcinogen are highly comparable to human colon tumors in their histological appearance as well as in their behaviour against chemotherapeutics. The development of such a new type of drug is of special interest because tumors of the colon account for a high percentage of cancer mortality today.

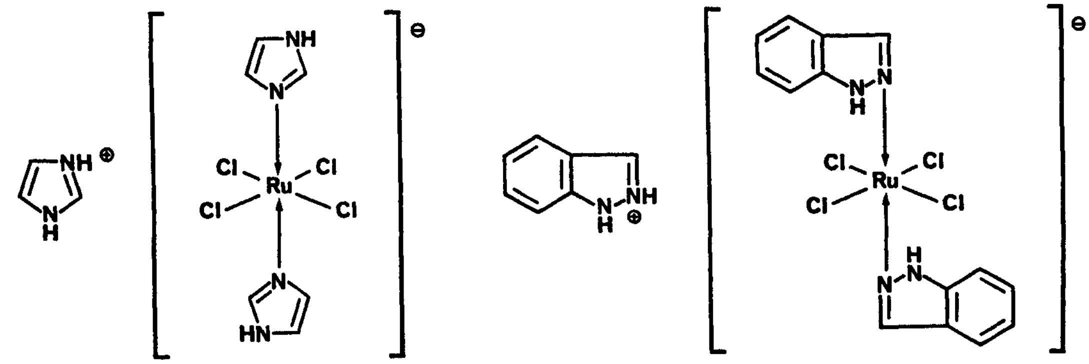

**Figure 1.** Structures of *trans*-HIm[$RuCl_4(im)_2$] **ICR** and *trans*-HInd[$RuCl_4(ind)_2$] **1**.

Extensive studies have been made into the fact that ruthenium complexes with good antitumor effects undergo numerous conversions *in vivo* and are transformed into the species which is active at the affected area by metabolic processes[1]. Therefore Ru(III) complexes are better handled as "prodrugs", which are converted *in situ* into the corresponding, more labile, species. These complexes are able to dissociate one or more non-nitrogen ligands, which in our case are the reactive $Cl^-$ leaving groups, to undergo bonding with the target molecule. It is supposed that in the first step $H_2O$-molecules coordinate at the free positions until bonding to a biomolecule. In this paper, the results of these investigations for complex **1** and its derivatives are reported in comparison with the hydrolysis of **ICR** as well as extensive studies into its synthesis. We discuss the structure of the analogous complex *trans*-H(1-MeInd)[$RuCl_4$(1-MeInd)$_2$] x 1$H_2O$ **6** and its hydrolysis product [$RuCl_3(H_2O)$(1-MeInd)$_2$] **7** with 1-methylindazole as organic ligand L.

### Materials and Methods

**Materials.** "$RuCl_3$ x 3 $H_2O$" was obtained from Degussa (Frankfurt, FRG) and was used after purification according to methods which are listed in the following section; indazole was provided by Degussa (Frankfurt / Main), and 1- and 2-methylindazole were produced by reduction of N-substituted o-nitrobenzylidenamine with lithium aluminium hydride according to the method of Bakke and Skjervold[15].

**General instructions.** The purification methods for "$RuCl_3$ x 3 $H_2O$" (production of pure Ru(III) solutions with different properties) were as follows:

Method I: Diluted ruthenium solution.
8 g (28.34 mmol) of $RuCl_3$ x 3 $H_2O$ were heated for one hour under reflux in 50 ml of 1 N HCl and ethanol (50 ml). After cooling the dark red solution down to 40°C, the alcohol and the produced acetaldehyde were removed under reduced pressure. The residues were filtered and filled up with 1 N HCl to give a volume of 100 ml. The solution thus obtained contains 28.3 mmol of ruthenium, which is present in the oxidation state +III exclusively.

Method II: Concentrated ruthenium solution.

The procedure is as described under method I, but instead of 1 N HCl concentrated HCl (12 N) was used.

Method III: Ruthenium solution in 1 N ethanolic HCl.
The procedure is as described under method I, but instead of 1 N HCl solution 1 N ethanolic HCl solution was used and preparation is carried out under nitrogen atmosphere. The alcoholic hydrochloric acid was produced by letting HCl gas stream through absolute ethanol. The concentration was determined by titration against 1 N NaOH.

**Complex Syntheses.** The preparation of the complexes with indazole, 1-, and 2-methylindazole as nitrogen ligands can be carried out by the following methods and is highly dependent on exact reaction conditions. The quantity of the heterobases varies according to the desired products and the used ligands. With indazole, five different ruthenium complexes could be isolated and characterized, two of them are new types not yet mentioned in the literature. Crystallization of the complexes was difficult because of hydrolysis in most of the suitable solvents. All new compounds gave satisfactory elemental analyses.

***trans*-HInd[RuCl$_4$(ind)$_2$] (1)** . General method: Indazole (6.0 g; 51 mmol) was dissolved in 80 ml of 12 N HCl at 70 °C. 20 ml of the Ru(III) solution (method II) were added to the hot solution and the mixture was stirred and heated for about 15 min at 80-90 °C. An ochre-colored solid separated from the solution almost immediately. Stirring was continued until ambient temperature was reached and then the crude material was separated and stirred with water for about two hours to remove the HCl residues. Then the solid was filtered and washed with ethanol and diethylether and dried *in vacuo*. To optimize the yield, the solution was stirred again for several hours at about 80-90°C. The purification of the separating solid was carried out as mentioned above. The yield of the microcrystalline product **1** was: 2.96 g (87 %) (decomp.: 248 °C; $\Lambda_M$= 44.0 [$\Omega^{-1}cm^2mol^{-1}$]). Anal. Calcd for $C_{21}H_{19}N_6Cl_4Ru$: C, 42.16; H, 3.20; N, 14.05; Cl, 23.70; Ru, 16.89. Found: C, 42.20; H, 3.35; N, 13.83; Cl, 23.84; Ru, 17.32. IR (cm$^{-1}$): ν(Ru-Cl) 322 s, 295 sh; ν(Ru-N) 283 s, 216 s; δ(N-Ru-Cl) 179 w; δ(Cl-Ru-Cl) 151 w.

When using the Ru(III) solution prepared according to method I, like in the synthesis of the analogous imidazole complex **ICR** and all other complexes of the HL[RuCl$_4$L$_2$]-type we have synthesized so far, the oxo complex **2** was formed instead of the intended complex **1**.

**(HInd)$_4$[Ru$_2$OCl$_8$(ind)$_2$] (2)** . General method: Indazole (6.0 g; 51 mmol) was dissolved in 8 N HCl at 0-5 °C and 20 ml of the Ru(III) solution (method I) were added with vigorous stirring. The colour of the mixture gradually turned into a deep red and after 10 min a dark violet solid began to precipitate. The reaction mixture was allowed to stand for two days at 5 °C and then the product was filtered, washed with $H_2O$ and dried over $P_2O_5$. The yield of **2** was: 2.44 g (34 %) (mp: 160-163 °C; $\Lambda_M$= 38.5 [$\Omega^{-1}cm^2mol^{-1}$]). Anal. Calcd for $C_{42}H_{40}N_{12}Cl_8ORu_2$: C, 41.53; H, 3.22; N, 13.84; Cl, 23.35; Ru, 16.64. Found: C, 41.63; H, 3.39; N, 13.57; Cl, 23.58; Ru, 15.75. IR (cm$^{-1}$): ν(Ru-O-Ru)$_{asym}$ 896 m; δ(Ru-O-Ru) 457 m; ν(Ru-Cl) 323 s, 293 s; ν(Ru-N) 271 s, 222 w; δ(O-Ru-Cl), δ(Cl-Ru-Cl) 194 w, 170 w.

HPLC investigations into complex **2** showed that the samples contained impurities of complex **1**. At room temperature, the share of **1** is between 10% and 25 %, but at low temperatures in the range between 0°C and 5 °C, the share of this competitive product could be kept below 8 %.

**(HInd)$_2$[RuCl$_5$(ind)] x 0.5 H$_2$O (3).** General method: Indazole (2.0 g; 17 mmol) was dissolved in 10 ml of 2 N HCl at a temperature of 60-70 °C. 20 ml of the Ru(III) solution (method II) were added to the warm solution. The red-brown solid precipitated immediately. However, if complex **1** is produced in a competitive reaction, which is observed as the separation of an ochre-colored solid, the precipitate must be dissolved again by adding approximately 10 ml of 12 N HCl. The mixture was then allowed to stand for 24 hours at room temperature. The brown-red product was washed with water, ethanol and diethylether and dried *in vacuo*. The yield of **3** was: 1.82 g (50 %) (decomp.: 208-210 °C; $\Lambda_M$= 54.2 [$\Omega^{-1}cm^2mol^{-1}$]). Anal. Calcd for $C_{21}H_{20}N_6Cl_5Ru$ x 0.5 $H_2O$: C, 39.18; H, 3.29; N, 13.05; Cl, 27.54; Ru, 15.70. Found: C, 39.23; H, 3.29; N, 13.19; Cl, 27.44; Ru, 15.48. IR (cm$^{-1}$): ν(Ru-Cl) 304 s, 270 sh; ν(Ru-N) 218 m.

**$(HInd)_4[Ru_2OCl_{10}]$ (4)**. General method: Indazole (1.30 g; 11 mmol) was dissolved in 90 ml of 1 N ethanolic HCl. 10 ml of the Ru(III) solution (method III) were added and the solution thus obtained was then allowed to stand for 24 hours at room temperature. The dark red crystals were filtered, washed with anhydrous ethanol and diethylether and dried *in vacuo*. The yield of **4** was: 0.93 g (31 %) (decomp.: 193 °C; $\Lambda_M$= 67.6 [$\Omega^{-1}cm^2mol^{-1}$]). Anal. Calcd for $C_{28}H_{28}N_8Cl_{10}ORu_2$: C, 32.05; H, 2.69; N, 10.68; Cl, 33.79; Ru, 19.27. Found: C, 32.12; H, 2.72; N, 10.64; Cl, 33.32. IR ($cm^{-1}$): $\nu(Ru\text{-}O\text{-}Ru)_{asym}$ 888 w; $\nu$(Ru-Cl) 347 m, 326 s, 312 sh; $\nu(Ru\text{-}O\text{-}Ru)_{sym}$ 265 sh; $\delta$(O-Ru-Cl), $\delta$(Cl-Ru-Cl) 201 w, 194 m.

**$(HInd)_3[RuCl_6]$ (5)**. General method: After dissolving indazole (1.15 g; 9.73 mmol) in 5 ml of 12 N HCl at 70 °C, 10 ml of the Ru(III) solution (method II) were added with vigorous stirring. The voluminous red-brown solid separated immediately, and after stirring until room temperature was reached, the mixture was allowed to stand for 24 hours at this temperature. The microcrystalline product was washed with $H_2O$, with an ethanol / diethylether mixture (1:1) and finally with diethylether and then dried over $P_2O_5/CaCl_2$. The yield of **5** was: 0.86 g (45 %) (decomp.: 170 °C; $\Lambda_M$= 58.9 [$\Omega^{-1}cm^2mol^{-1}$]). Anal. Calcd for $C_{21}H_{21}N_6Cl_6Ru$: C, 37.58; H, 3.15; N, 12.52; Cl, 31.69; Ru, 15.06. Found: C, 37.37; H, 3.14; N, 12.34; Cl, 30.70. IR ($cm^{-1}$): $\nu$(Ru-Cl) 305 vs.

***trans*-H(1-MeInd)[$RuCl_4$(1-MeInd)$_2$] x 1$H_2O$ (6)**. General method: 1-Methylindazole[15] (0.9 g; 6.81 mmol) was dissolved in 3.4 ml of 8 N HCl. Then 8.2 ml of the Ru(III) solution (method I) were added with stirring. The solution was filtered to remove undissolved residues and after 24 hours the voluminous ochre-coloured complex **6** was filtered and washed with $H_2O$ and diethylether. The product **6** was dried over $P_2O_5$. Yield: 1.15 g (75 %) (mp: 216-219 °C; $\Lambda_M$= 32.0 [$\Omega^{-1}cm^2mol^{-1}$]). The crude crystals were recrystallized from an acetone / ethanol solution (1:1). X-ray crystallographic data is listed in Table 1. Anal. Calcd for $C_{24}H_{25}N_6Cl_4Ru$ x 1$H_2O$: C, 43.78; H, 4.13; N, 12.76; Cl, 21.54; Ru, 15.34. Found: C, 43.72; H, 3.93; N, 12.73; Cl, 21.96; Ru, 14.81. IR ($cm^{-1}$): $\nu$(Ru-Cl) 335 s, 306 sh; $\nu$(Ru-N) 242 w.

***trans*-[$RuCl_3(H_2O)$(1-MeInd)$_2$] (7)**. General method: Complex **6** (0.1 g; 15.2 mmol) was dissolved in 40 ml of an acetone / water mixture (1:1). The solution was filtered to remove undissolved residues, and after two days red microcrystals were grown by slow evaporation at room temperature. The yield of **7** was: 56 mg (75 %) (decomp.: 260 °C; $\Lambda_M$= 1.2 [$\Omega^{-1}cm^2mol^{-1}$]). X-ray crystallographic data is listed in Table 1. Anal. Calcd for $C_{16}H_{18}N_4Cl_3ORu$: C, 39.24; H, 3.70; N, 11.44; Cl, 21.72; Ru, 20.64. Found: C, 39.50; H, 3.75; N, 11.40; Cl, 21.03. IR ($cm^{-1}$): $\nu(H_2O)_{sym}$ 3390 s,b; $\nu(H_2O)_{asym}$ 3247 vs; $\nu(Ru\text{-}OH_2)$ 410 w, 856 ms; $\nu$(Ru-Cl), $\nu(Ru\text{-}OH_2)$ 340 s,b, 325 sh; $\nu$(Ru-N) 230 w.

**(1-MeInd)$_4$[$Ru_2OCl_{10}$] x 2HCl (8)**. General method as for complex **6**, but with concentrated Ru(III) solution (method II). The brown-red complex precipitated immediately. The yield of **8** was: 1.05 mg (38 %) (decomp.: 200-203 °C; $\Lambda_M$= 154.5 [$\Omega^{-1}cm^2mol^{-1}$]). The initial dark orange solution of **8** decomposes after a few minutes in water and in methanol to give a colourless solution. Anal. Calcd for $C_{32}H_{36}N_8Cl_{10}ORu_2$ x 2HCl: C, 32.62; H, 3.25; N, 9.51; Cl, 36.11; Ru, 17.16. Found: C, 32.73; H, 3.20; N, 9.41; Cl, 35.70; Ru, 16.28. IR ($cm^{-1}$): $\nu(Ru\text{-}O\text{-}Ru)_{asym}$ 899 w; $\delta$(Ru-O-Ru) 470 m; $\nu$(Ru-Cl) 298 vs, 314 sh; $\nu(Ru\text{-}O\text{-}Ru)_{sym}$ 251 w; $\delta$(O-Ru-Cl), $\delta$(Cl-Ru-Cl) 202 w, 192 m.

**(2-MeInd)$_4$[$Ru_2OCl_{10}$] x 2HCl (9)**. General method: 2-Methylindazole[15] (1.0 g; 7.6 mmol) was dissolved in 8 N HCl, and 8.8 ml of the Ru(III) solution (method I) were added at room temperature. The solution turned into a dark brown, and after adding 2 ml of 12 N HCl and heating the mixture to 80-90 °C, a powdery solid began to separate. The product was carefully washed with water and dried over $P_2O_5/CaCl_2$. The yield of **9** was: 1.66 mg (56 %) (decomp.: 220-223 °C; $\Lambda_M$= 153.0

[$\Omega^{-1}cm^2mol^{-1}$]). There was no reaction with the preparation method of the 1-methyl-complex (**6**). The addition of concentrated HCl and a higher reaction temperature is necessary in the synthesis of complex **9**. Anal. Calcd for $C_{32}H_{36}N_8Cl_{10}ORu_2$ x 2HCl: C, 32.62; H, 3.25; N, 9.51; Cl, 36.11; Ru, 17.16. Found: C, 32.80; H, 3.40; N, 9.13; Cl, 35.89. IR ($cm^{-1}$): $\nu(Ru\text{-}O\text{-}Ru)_{asym}$ 901 m; $\delta$(Ru-O-Ru) 470 m; $\nu$(Ru-Cl) 298 vs,b; $\nu(Ru\text{-}O\text{-}Ru)_{sym}$ 251 w; $\delta$(O-Ru-Cl), $\delta$(Cl-Ru-Cl) 201 w, 191 m.

**Physical measurements.** Electronic spectra were recorded on a Perkin Elmer Lambda 9 UV/VIS/NIR spectrophotometer in connection with a Perkin Elmer 7300 Professional Computer. Infrared spectra were recorded with CsI-pellets on a Perkin Elmer 983 G infrared spectrophotometer or a Bruker IFS 113 for spectra in the far IR region. The $^1H$ NMR spectra were taken on a Bruker Ac 200 MHz spectrophotometer and a Bruker AMX 500 MHz spectrophotometer at a temperature of 293 K. Dimethyl-$d_6$-sulfoxide was used as solvent. Conductivity measurements were carried out using an LF191 (WTW) digital conductometer with an LS1/T-1.5 platinum electrode in dimethylformamide (DMF). The cell constant was calibrated to 12.88 mS with 0.1 M KCl solution in bidistilled water. Measurements were carried out with 0.001 molar solutions at a temperature of 298 K in a water bath. Solvent conductivities were 0.003-0.005 $mScm^{-1}$ for DMF and 0.001-0.003 $mScm^{-1}$ for $H_2O$ (bidistilled). HPLC analyses were carried out at 295 K with a Latek P-400 pump, a Merck-Hitachi L-3000 photo-diode-array detector and a Merck-Hitachi chromato-D-2000 integrator. A nucleosil (300 Å / 7$\mu$; Macherey-Nagel, Düren, FRG) diole column (250 mm x 8 mm) was used for chromatography. The mobile phase used consisted of 70 % acetonitrile (Lichrosolv; Merck, Darmstadt, FRG) and 30 % 5 mM $KH_2PO_4$ (pa; Merck, Darmstadt, FRG) in bidistilled water, pH = 4.5. The flow rate was 3 ml/min and UV detection was carried out at 210 nm. The volume of samples injected onto the column was 20 $\mu$l.

**X-Ray Crystal Structure Determination.** Structures were determined on a Siemens-Stoe AED II - Diffractometer[16] at room temperature using *Mo K$\alpha$* radiation ($\lambda$ = 0.71073 Å) and a graphite monochromator. Crystal structures were solved by the SHELXTL PLUS program[17] and Patterson and Fourier methods. Refinement parameters were calculated by the full-matrix-least-squares method and the atomic form factors were taken from the International Tables[18]. Crystallographic data for **6** and its aqua complex **7** is summarized in Table 1, atomic positional parameters, bond lengths and angles are given in Tables 2 and 4 for **6** and in Tables 3 and 5 for **7**. The structures of **6** and **7** are shown in Figures 7 and 8.

### Results and Discussion

**Investigations into the synthesis of the complex $HInd^+[RuCl_4(ind)_2]^-$ (1).** Considering the antitumor activity of the complex **ICR** against tumors of the colon and rectum, which has already been tested with good success, the analogous indazole complex **1** is of major interest for clinical use because of its even higher activity. After the failure to produce complex **1** in the usual way that was used in the case of the complexes of the $HL[RuCl_4L_2]$-type we have synthesized so far and which have already been described in earlier publications[3,14], the reaction procedures for indazole and Ru(III) solutions were intensively studied. First we copied the synthesis for the imidazole complex **ICR** and dissolved indazole in 8 N HCl while slightly heating and, after cooling down, the diluted Ru(III) solution (method I) was added to the solution with stirring. Then the mixture was examined over 48 hours by means of HPLC measurements. After ten minutes, the first sample was taken and immediately put onto the column. The procedure was repeated every ten minutes over a period of 6 hours. The indazolium cation ($HInd^+$), which is predominant, could be detected at a retention time of about 3 minutes. Besides, three other product peaks could also be detected. Their increasing percentages throughout the reaction were determined by peak areas (Fig. 2). The composition of the reaction mixture measured by HPLC after 24 h showed that three ruthenium complexes are formed in different shares: A (10 %), B (57 %) and C (33 %).

In a second experiment, 2 mg of the solid which was isolated from the reaction mixture after 24 hours by filtration, was dissolved in $H_2O$ bidest. and separated under the usual conditions on an analytical column. The HPLC chromatogram of the isolated solid is shown in Fig. 3. The percentage composition is in good agreement with that of the liquid phase.

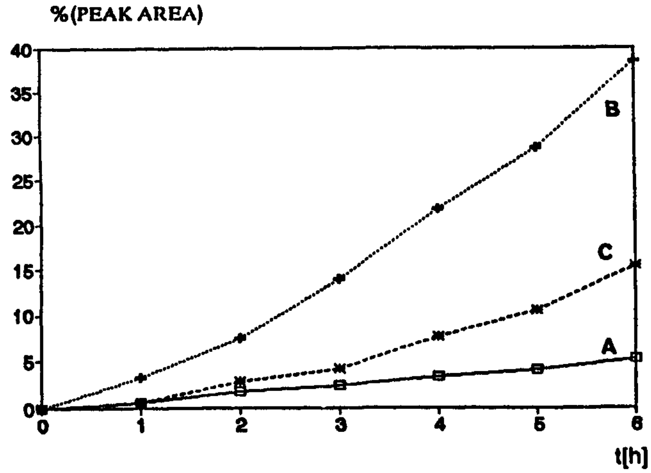

**Figure 2.** Percentage increase of products A, B, and C during the reaction of Ru(III) solution with indazole in 8 N HCl. (A = $[RuCl_4(ind)_2]^-$; B = $[Ru_2OCl_8(ind)_2]^{4-}$; C = $[Ru_2OCl_6(H_2O)_2(ind)_2]^{2-}$).

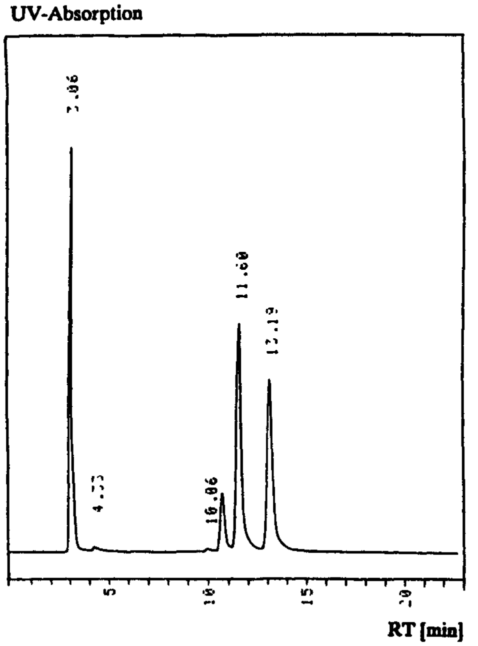

**Figure 3.** HPLC of the solid which was isolated from the reaction mixture after 24 hours. Column: nucleosil/diole 300 Å/7μ; eluent: 70 % $CH_3CN$ / 30 % 5 mmol $KH_2PO_4$. Dimensions: 250 x 8 mm; Flow: 3 ml/min.; Detection: UV, 210 nm. (A = $[RuCl_4(ind)_2]^-$; B = $[Ru_2OCl_8(ind)_2]^{4-}$; C = $[Ru_2OCl_6(H_2O)_2(ind)_2]^{2-}$). RT = 3.06→ $HInd^+$, RT = 10.06→ A, RT = 11.60→ B, RT = 13.19→ C.

The different fractions were collected, the solvent removed under reduced pressure, dried *in vacuo*, and then observed by means of UV-, IR- and $^1H$ NMR spectroscopy. By making a comparison with the IR spectra of pure $KH_2PO_4$, the IR absorptions caused by residues of the added buffer could be assigned. The $^1H$ NMR measurements were carried out in $D_2O$ or in $[d_6]$DMSO to distinguish between the presence and absence of an aqua complex. The signal at a retention time of about three minutes can undoubtedly be assigned to the $HInd^+$ cation by means of comparative UV measurements with free indazole. Also, the $^1H$ NMR of this fraction shows the characteristic chemical shifts of the indazolium cation $HInd^+$ ($\delta$ = 8.04[s, 1H, 3-H]; 7.74[d, 1H, 4-H]; 7.52[d, 1H, 4-H]; 7.33[t, 1H, 5-H]; 7.09[t, 1H, 6-H]. As expected, no highfield-shifted signals caused by anionic ruthenium-bonded species can be observed.

The following structures could be resolved for the complexes A, B and C:

A : $[RuCl_4(ind)_2]^-$ (anion of complex **1**)

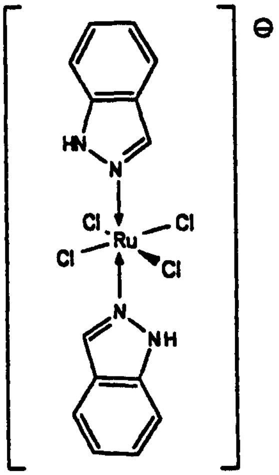

B : $[Ru_2OCl_8(ind)_2]^{4-}$ (anion of complex **2**)

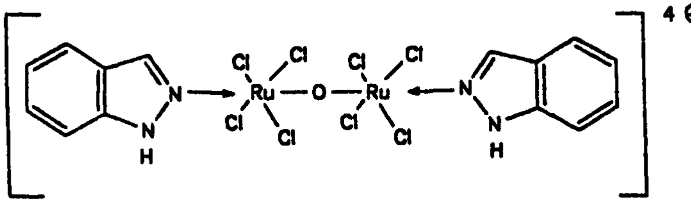

C : $[Ru_2OCl_6(H_2O)_2(ind)_2]^{2-}$

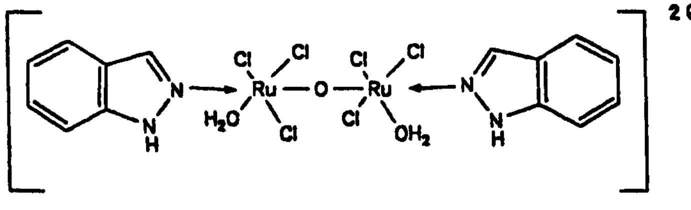

A comparison of the IR spectra and the UV spectra in anhydrous methanol makes it definite that the component C is not present in the isolated solid, but is produced out of the bridged oxo-complex B in water by substitution of the $Cl^-$ anions: there are no IR absorptions, which should be registered if an aqua complex existed, and the UV spectra show no evidence of the presence of component C. Only if water is added do the UV spectra of the solid product and the reaction solution become identical. Besides these facts, the reproducibly constant results of the analytical data prove the composition of the mixture "ABC" to consist of 10-25 % A: $HInd^+[RuCl_4(ind)_2]^-$ (**1**)

and 75-90 % B: $(HInd^+)_4[Ru_2OCl_8(ind)_2]^{4-}$ **(2)**. If the bis-aqua complex C: $(HInd^+)_2[Ru_2OCl_6(H_2O)_2(ind)_2]^{2-}$ was present in the solid with an average share of at least 30 %, which is indicated by HPLC experiments, the calculated value for carbon would be 1-1.5 % higher and the nitrogen value would be about 0.5 % lower.

Finally we succeeded in transforming the product mixture into the pure complex **1** by treating it with concentrated HCl at a raised reaction temperature. For that, 60 ml of 12 N HCl in portions of 10 ml were given to the reaction mixture over a period of 6 hours. A sample for HPLC was taken each time before adding the acid. The addition of the acid lead to a dramatic change in the composition of the solution. The results are summarized in Fig. 4.

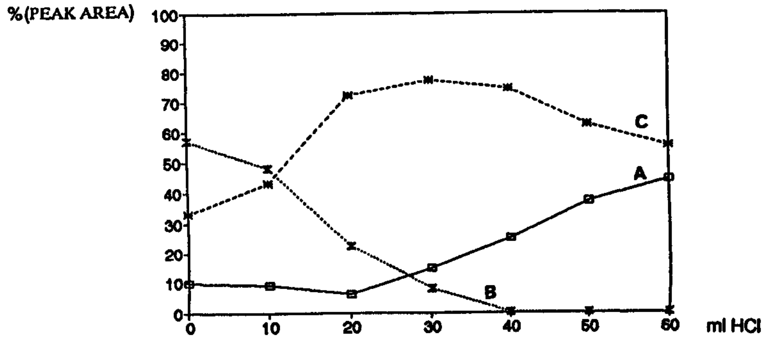

**Figure 4.** Difference in percentage composition of the reaction solution of complex **1** with addition of conc. HCl. (A = $[RuCl_4(ind)_2]^-$; B = $[Ru_2OCl_8(ind)_2]^{4-}$; C = $[Ru_2OCl_6(H_2O)_2(ind)_2]^{2-}$).

After adding 10 ml of HCl, the concentration of the oxo-bridged species, which had been predominant in the violet solution up to this point, decreased. The percentage composition after

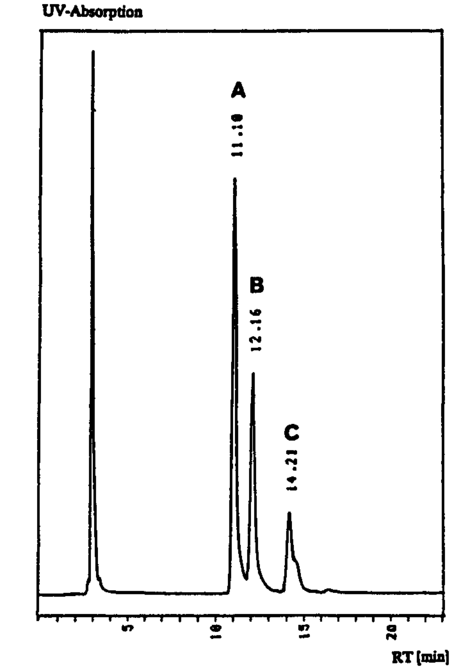

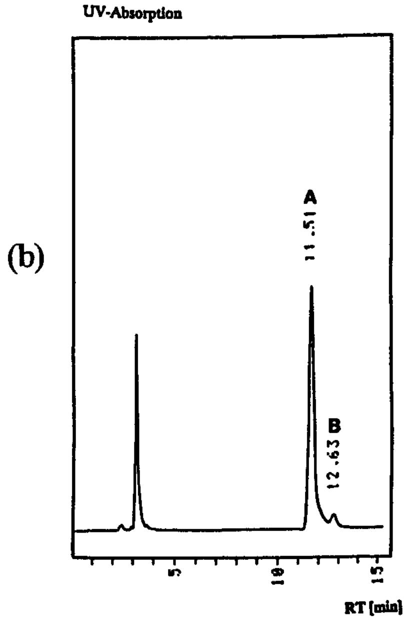

**Figure 5a.** HPLC of the isolated product after the addition of conc. HCl (A = anion of **1**). (A = $[RuCl_4(ind)_2]^-$; B = $[Ru_2OCl_8(ind)_2]^{4-}$; C = $[Ru_2OCl_6(H_2O)_2(ind)_2]^{2-}$). The peak at RT = ca. 3 min. is $HInd^+$.

**Figure 5b.** HPLC of the isolated product after further treatment with conc. HCl (A = anion of **1**). (A = $[RuCl_4(ind)_2]^-$; B = $[Ru_2OCl_8(ind)_2]^{4-}$). The peak at RT = ca. 3 min. is $HInd^+$.

dropping 20 ml of the acid into the reaction mixture leaves merely 8 % of this product. In contrast, the concentration of the desired complex **1** increased and after the addition of 60 ml of HCl it prevailed in the solution, which at that time had a brown colour. At the beginning of the addition the aqua complex C reacted with a slight increase in its concentration, but when further raising the $Cl^-$ concentration, its formation becomes increasingly hindered. This could be seen in further HPLC investigations. The beige-coloured solid which had formed throughout the addition of the 12 N HCl was separated by filtration, washed with $H_2O$ and ethanol / diethylether (1:1) and dried *in vacuo*. The HPLC of this product is shown in Fig. 5a. It is evident that complex **1** provides the main product after the treatment with concentrated HCl. Disturbing residues of the oxo-bridged complex **2** and the aqua complex C, which forms out of **2**, could be avoided by further treatment of the solid product with 6 N HCl at 90 °C. A HPLC chromatogram of this product after usual purification shows nearly complete transformation (Fig. 5b). It could be proved that complex **1** can be made available from the oxo complex **2** by raising the temperature and adding concentrated HCl. This knowledge lead us to the general method for the synthesis of **1**, which turns out in a remarkably high purity and which is described in the section dealing with complex synthesis.

**Bonding in complex 1.** Bonding conditions in complex **1** were elucidated by studying the interactions between ruthenium(III) and the two N-methyl isomers of indazole. In a neutral or acid solution, the indazole molecule exists in a mesomer equilibrium: the five-membered ring of the indazole can achieve bonding to the metal with its N-1 or with the N-2 nitrogen. To clarify the situation, both N-methyl derivatives were synthesized in high purity according to the method of Bakke and Skjervold[15]. While in the case of the 1-methyl derivative the complex *trans*-H(1-MeInd)[$RuCl_4$(1-$MeInd_2$)] **(6)** could easily be prepared according to our usual method for complexes of the HL[$RuCl_4L_2$]-type, the 2-methylindazole did not show any tendency towards binding to the metal, even if the parameters of the reaction (molar proportions, temperature, pH, time of reaction) were altered. Only the oxo-species $(2\text{-MeIndH})_4[Ru_2OCl_{10}]$ x 2 HCl **(9)** could be obtained when a concentrated Ru(III) solution was used. So it can be expected that the unsubstituted indazole in complex **1** is linked to the ruthenium through the N2-nitrogen.

**HPLC investigations into the solution chemistry of 1 in comparison with ICR.** HPLC is an important method to prove the purity of a new drug before it can undergo clinical studies. We investigated the hydrolysis reactions of the two active compounds **1** and **ICR**, and the newly formed hydrolysis products were examined. Stability of the complexes in physiological saline was observed by means of HPLC over a period of two days and by conductivity measurements. While the decomposition of **ICR** is about 4 % per hour, that of $[RuCl_4(ind)_2]^-$ **1** is less than 0.9 % per hour. The pseudo-first-order rate constant of hydrolysis of **ICR** at 22 °C was determined to be $9.10 \times 10^{-4}\ s^{-1}$ and for complex **1**, it is $1.55 \times 10^{-4}\ s^{-1}$. Half-lifes were calculated to be 12.7 hours in the case of **ICR** and 74.3 hours in the case of **1**. These results are in agreement with experiments carried out under slightly changed conditions[19-20].

The hydrolysis of **ICR** causes the formation of three new products while in the case of the indazole complex there is only one. The hydrolysis products were analysed by comparative HPLC measurements, UV spectroscopy and conductivity measurements. The main product of the hydrolysis of **ICR** is the neutral complex $[RuCl_3(im)_3]$, which appears at a retention time of about three minutes. This could be proved by comparative UV measurements with the pure trisimidazole complex, which we had already been able to synthesize[21]. UV detection was carried out at 336 nm, because the characteristic absorption of $[RuCl_3(im)_3]$ in water is expected at this value. Under the experimental conditions in the HPLC measurements (mobile phase: 70 % acetonitrile / 30 % 5 mmol $KH_2PO_4$), this band is shifted to 346 nm in the case of $[RuCl_3(im)_3]$ as well as for the HPLC-fraction at about three minutes retention time. After 24 hours the trisimidazole complex is the major hydrolysis product in the solution with a share of about 60 %. The two other hydrolysis products are likely to be aqua complexes having as they do an absorption band at about 360 nm, which is characteristic of the $Cl^-$ substitution in the complex transformation from *trans*-H(1-MeInd)[$RuCl_4$(1-$MeInd_2$)] x $1H_2O$ **6** to the aqua species [$RuCl_3(H_2O)$(1-MeInd)$_2$] **7**. The composition of the **ICR** solution after 5 min, 12 hours and 24 hours is shown in Fig. 6.

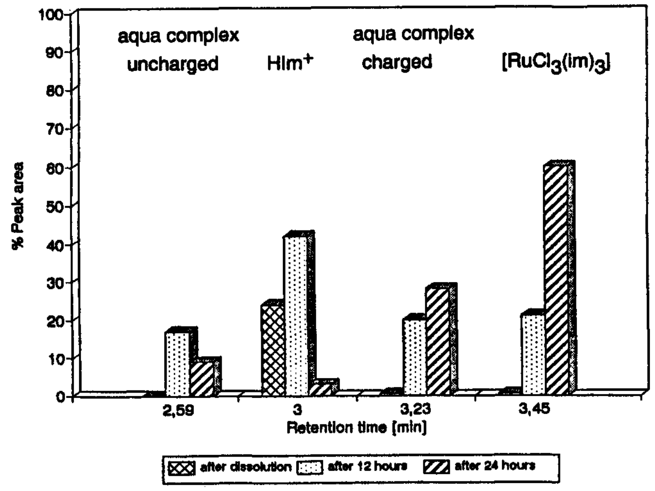

**Figure 6.** Peak areas in the HPLC experiment of the four hydrolysis products of ICR after dissolution, 12 and 24 hours. (The intact $[RuCl_4(im)_2]^-$ is not shown.)

The concentration of the cation $HIm^+$ decreases in accordance with the formation of the tris-imidazole complex. The ionic aqua complex $[RuCl_2(H_2O)_2(im)_2]^+$ is expected at a higher retention time than the neutral aqua species $[RuCl_3(H_2O)(im)_2]$. On the other hand, a characteristic feature of the hydrolysis of the indazole complex **1** is that no trisindazole complex is formed. This could be confirmed by experimental work. The trisindazole complex could not be obtained, not even through modification of the synthesis of the analogue $[RuCl_3(im)_3]$. This fact seems to be important in view of the remarkably high stability of **1** in the hydrolysis experiments. The HPLC investigations into this complex have shown that only one product is formed over a period of 10 hours. The evaluation of UV- and conductivity measurements suggests that this product is probably an aqua complex.

**UV spectroscopy and conductivity measurements.** In dimethylformamide, **ICR** equals a 1:1 electrolyte with "slow" cations and anions ($\Lambda_M$ = 52 $\Omega^{-1}cm^2mol^{-1}$ at 25 °C). Dissolved in water (pH = 6.0), it also equals a 1:1 electrolyte, with the molar conductivity $\Lambda_M$ increasing over the time of measurement from 68 $\Omega^{-1}cm^2mol^{-1}$ to 143 $\Omega^{-1}cm^2mol^{-1}$. This increase can be attributed to a relatively slow exchange of a $Cl^-$ ion for $H_2O$ and the simultaneous formation of an aqua complex. Since $Cl^-$ itself has a higher conductivity than the complex anion, the molar conductivity increases:

$$HIm^+[RuCl_4(im)_2]^- + H_2O \longrightarrow [RuCl_3(H_2O)(im)_2] + HIm^+ + Cl^-$$

However, prolonged observation periods over 2 days showed that conductivity still increases very slowly up to values in the range of 2:1 electrolytes. This can be attributed to the formation of another aqua complex, with two chloride ions replaced by water. The formation of a trisimidazole complex, which is responsible for the lowered pH of 4.2 of **ICR** in aqueous solution, could be a second possibility and is in good agreement with the results of the HPLC investigations:

$$HIm^+[RuCl_4(im)_2]^- + H_2O \longrightarrow [RuCl_3(im)_3] + H_3O^+ + Cl^-$$

The molar conductivity of the isolated neutral trisimidazole complex is 0.8 $\Omega^{1}cm^{2}mol^{-1}$ in DMF and 9.9 $\Omega^{-1}cm^{2}mol^{-1}$ in water.

The formation of compound $[RuCl_3(im)_3]$ is further elucidated by UV measurements over a hydrolysis period of 14 hours at ambient temperature. We observed UV absorption directly after dissolving **ICR** in water and during 14 hours of hydrolysis respectively. When comparing the results with the absorption spectrum of $[RuCl_3(im)_3]$ in water, the transformation can be seen. The absorption at 347.4 nm, which is characteristic of **ICR**, is continuously shifted to 336 nm, which is identical with the characteristic absorption band of the pure trisimidazole complex.

As expected, the molar conductivity of the complex $HInd^{+}[RuCl_4(ind)_2]^{-}$ (**1**) in DMF equals a 1:1 electrolyte (44 $\Omega^{-1}cm^{2}mol^{-1}$). This value remained almost constant over 24 hours. When the complex is dissolved in water, it shows, from the very beginning, high molar conductivity (307 $\Omega^{-1}cm^{2}mol^{-1}$), which increases very slightly to 360 $\Omega^{-1}cm^{2}mol^{-1}$ after 24 hours.

**Molecular structures of the complexes 6 and 7.** The initial formation of aqua complexes in the hydrolysis reaction of complex type $HL[RuCl_4L_2]$ could be confirmed by the isolation of complex **7**, which is produced from complex **6** dissolved in an aqueous medium. We could crystallize both compounds and resolve the X-ray crystal structures, which are shown in Figs. 7 and 8. Because of the fact that the aqua species **7** is formed out of complex **6** in a hydrolysis reaction in which a $H_2O$ molecule is substituted for a $Cl^-$ ligand, a comparative examination of these two compounds seems reasonable. In both cases, the ruthenium is placed in the center of a slightly distorted octahedron, in which the two *trans*-standing 1-methylindazoles are arranged in the axial positions (**6**: N(1)-Ru(1)-N(2): 179.8°; **7**: N(2)-Ru(1)-N(2a): 174.3°), and the four $Cl^-$ atoms in complex **6** are in the equatorial positions, which in the case of complex **7** are occupied by three $Cl^-$ atoms and the $H_2O$ molecule. Bonding to the metal is via the N2-nitrogen of the organic ligand. Compound **6** crystallizes with four molecules in the unit cell of space group P2.1/n, while in the aqua species **7** the four molecules are in a unit cell of space group C2/c (see Tables 1 and 2).

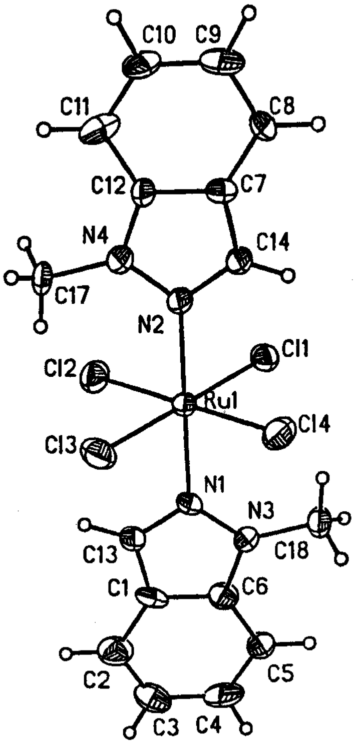

**Figure 7.** Molecular structure of the complex anion $[RuCl_4(1\text{-}MeInd)_2]^-$ of **6**.

**Table 1.** Crystal Data, Data Collection and Refinement Parameters for *trans*-H(1-MeInd)-$[RuCl_4(1\text{-}MeInd)_2]$ (**6**) and *trans*-$[RuCl_3(H_2O)(1\text{-}MeInd)_2]$ (**7**)

| complex | **6** | **7** |
|---|---|---|
| formula | $C_{24}H_{25}N_6Cl_4Ru$ x $1H_2O$ | $C_{16}H_{18}N_4Cl_3ORu$ |
| *fw* | 686.46 | 489.83 |
| space group | *P2.1/n* | *C2/c* |
| unit cell: | *a*: 10.511 | *a*: 19.90 |
| *a, b, c* [Å], β [°] | *b*: 13.87, β : 98.17 | *b*: 10.94, β : 96.74 |
| | *c*: 19.93 | *c*: 8.490 |
| volume [Å$^3$] | 2876.1 | 1835.6 |
| *Z* | 4 | 4 |
| density [g/cm$^3$] | 1.58 | 1.77 |
| *F*(000) | 1396 | 980 |
| crystal size [mm] | 0.6 x 0.6 x 0.95 | 0.05 x 0.1 x 0.35 |
| crystal system | monoclinic | monoclinic |
| absorption μ [cm$^{-1}$] | 9.4 | 1.29 |
| radiation, *MoKα* | λ = 0.71073 | λ = 0.71073 |
| scan technique | - | Θ/2Θ |
| max. 2C [°] | 48.5 | 50 |
| *h, k, l*-range | +13, +17, ±24 | ±23, +12, +10 |
| observed reflections | 4782 | 1709 |
| reflections with *I*>2.5σ *(I)* | 2407 | 770 |
| No. of parameters | - | 69 |
| *R, Rw* | 0.053, 0.048 | 0.059, 0.048 |
| goodness of fit | 1.75 | 1.56 |
| residual electron density [e/Å$^3$] | 1.48 | 1.69 |

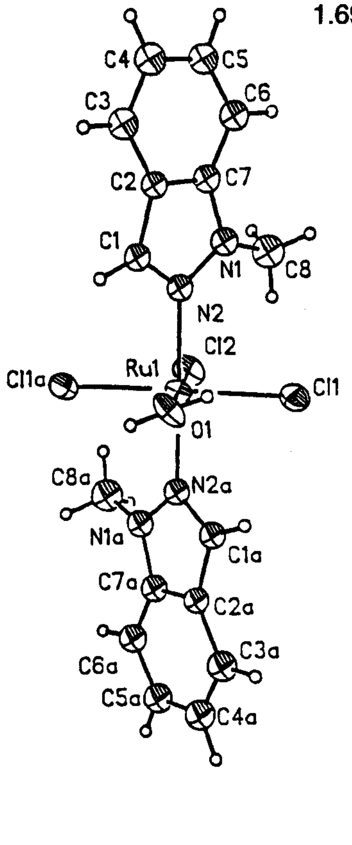

**Figure 8.** Molecular structure of **7**.

**Table 2.** Atomic Positional Parameters (x10[4])[a] and Equivalent Isotropic Displacement Parameters (x 10[3]) of complex **6**

| atom | *x* | *y* | *z* | $B_{eq}$(Å$^2$)[a] |
|---|---|---|---|---|
| Ru(1) | 4950(1) | 1863(1) | 2345(1) | 33(1) |
| Cl(1) | 4094(2) | 2979(2) | 1496(1) | 45(1) |
| Cl(2) | 4439(3) | 613(2) | 1518(1) | 52(1) |
| Cl(3) | 5794(2) | 754(2) | 3189(2) | 56(1) |
| Cl(4) | 5457(2) | 3097(2) | 3151(1) | 53(1) |
| N(1) | 6770(6) | 1928(6) | 2022(4) | 38(3) |
| N(2) | 3131(6) | 1799(6) | 2670(4) | 37(3) |
| N(3) | 7460(8) | 2715(6) | 1843(4) | 37(3) |
| N(4) | 2448(7) | 1013(6) | 2852(4) | 39(3) |
| C(1) | 8570(9) | 1389(9) | 1658(5) | 41(4) |
| C(2) | 9625(10) | 868(9) | 1485(6) | 56(5) |
| C(3) | 10592(10) | 1390(10) | 1261(6) | 54(5) |
| C(4) | 10548(10) | 2418(10) | 1227(6) | 62(6) |
| C(5) | 9541(9) | 2943(8) | 1399(5) | 45(4) |
| C(6) | 8551(10) | 2407(8) | 1619(5) | 42(5) |
| C(7) | 1314(9) | 2323(8) | 3032(5) | 35(4) |
| C(8) | 268(9) | 2836(7) | 3211(5) | 42(4) |
| C(9) | -723(10) | 2309(10) | 3411(6) | 55(5) |
| C(10) | -666(10) | 1302(10) | 3452(6) | 56(5) |
| C(11) | 329(10) | 764(9) | 3283(5) | 54(5) |
| C(12) | 1349(9) | 1315(8) | 3075(5) | 35(4) |
| C(14) | 2461(9) | 2570(7) | 2772(5) | 39(4) |
| C(13) | 7433(9) | 1134(8) | 1907(5) | 40(4) |
| C(17) | 2754(10) | 3(7) | 2734(6) | 58(5) |
| C(18) | 7143(9) | 3713(7) | 1981(6) | 55(5) |
| N(5) | 2258(10) | 7992(10) | 275(5) | 74(5) |
| N(6) | 3238(10) | 8292(11) | -61(5) | 89(6) |
| C(21) | 2251(15) | 7041(14) | 252(7) | 83(7) |
| C(22) | 1412(15) | 6394(16) | 503(8) | 108(9) |
| C(23) | 1588(23) | 5466(19) | 396(11) | 141(13) |
| C(24) | 2592(24) | 5081(19) | 62(11) | 157(15) |
| C(25) | 3402(18) | 5739(18) | -191(9) | 121(11) |
| C(26) | 3220(14) | 6707(15) | -83(7) | 82(7) |
| C(27) | 3867(13) | 7575(13) | -271(7) | 85(7) |
| C(28) | 1463(13) | 8710(12) | 538(7) | 125(9) |
| C(31) | 2512(15) | 1823(14) | -194(8) | 141(11) |
| C(32) | 3032(22) | 901(16) | -435(10) | 171(15) |
| O(33) | 3455(16) | 232(10) | -5(7) | 200(9) |

[a]the estimated standard deviations are shown in parentheses.

The Cl-Ru-Cl angles in the complex anion of **6** are 179.8° (Cl(1)-Ru(1)-Cl(3)) and 179.6° (Cl(2)-Ru(1)-Cl(4)), which differs only negligibly from linearity considering the Esd's. In the case of the neutral species **7**, only one angle with 180° could be observed (Cl(2)-Ru(1)-O(1)). A remarkable deviation from linearity could be observed for the angle Cl(1)-Ru(1)-Cl(1a): 172.6°, which is forced by steric hindrance of the methyl groups. It can be assumed that the Cl$^-$-atoms Cl(1) and Cl(1a), which approach the $H_2O$ molecule, form H-bridges between the protons of the $H_2O$ and the free electron pairs of the neighbouring chlorines, which would stabilize the aqua complex **7**. But this cannot really be proved with certainty because there is only a slight increase in the bond lengths (Ru-Cl(1) and Ru-Cl(1a): 2.356 Å) compared to the Ru-Cl(2) bond (2.304 Å), which is not in' olved in H-bridging.

**Table 3.** Atomic Positional Parameters (x $10^4$)[a] and Equivalent Isotropic Displacement Parameters (x $10^3$) of complex **7**

| atom | *x* | *y* | *z* | $B_{eq}$(Å$^2$)[a] |
|---|---|---|---|---|
| Ru(1) | 0 | 3201(2) | 2500 | 29(1) |
| Cl(1) | 663(2) | 3340(4) | 382(3) | 49(1) |
| Cl(2) | 0 | 1095(5) | 2500 | 49(2) |
| O(1) | 0 | 5129(15) | 2500 | 52(6) |
| N(1) | -1039(5) | 2657(9) | -507(11) | 40(3) |
| N(2) | -895(4) | 3297(10) | 911(10) | 37(3) |
| C(1) | -1402(6) | 4025(11) | 1122(14) | 36(3) |
| C(2) | -1915(6) | 3891(11) | -232(14) | 35(3) |
| C(3) | -2582(7) | 4356(12) | -608(15) | 47(4) |
| C(4) | -2970(7) | 3901(12) | -1954(15) | 49(4) |
| C(5) | -2710(6) | 3041(13) | -2846(14) | 47(4) |
| C(6) | -2072(6) | 2579(11) | -2546(14) | 45(4) |
| C(7) | -1680(6) | 2999(12) | -1162(13) | 39(3) |
| C(8) | -677(6) | 1642(12) | -1034(15) | 59(4) |

[a]the estimated standard deviations are shown in parentheses.

**Table 4.** Selected Bond Distances (Å) and Angles (deg) of complex **6**; Not including the disordered cation.

| Bonds | Length[a] | Bonds | Angles[a] |
|---|---|---|---|
| Ru(1)-Cl(1) | 2.373 (3) | Cl(1)-Ru(1)-Cl(2) | 87.5(1) |
| Ru(1)-Cl(2) | 2.400 (3) | Cl(1)-Ru(1)-Cl(3) | 179.8(1) |
| Ru(1)-Cl(3) | 2.359 (3) | Cl(2)-Ru(1)-Cl(3) | 92.5(1) |
| Ru(1)-Cl(4) | 2.355 (4) | Cl(1)-Ru(1)-Cl(4) | 92.2(1) |
| Ru(1)-N(1) | 2.105 (7) | Cl(2)-Ru(1)-Cl(4) | 179.6(1) |
| Ru(1)-N(2) | 2.107 (7) | Cl(3)-Ru(1)-Cl(4) | 87.8(1) |
| N(1)-N(3) | 1.385 (12) | Cl(1)-Ru(1)-N(1) | 91.4(2) |
| N(1)-C(13) | 1.341 (13) | Cl(2)-Ru(1)-N(1) | 86.9(2) |
| N(2)-N(4) | 1.380 (12) | Cl(3)-Ru(1)-N(1) | 88.8(2) |
| N(2)-C(14) | 1.313 (13) | Cl(4)-Ru(1)-N(1) | 92.9(2) |
| N(3)-C(6) | 1.357 (14) | Cl(1)-Ru(1)-N(2) | 88.8(2) |
| N(3)-C(18) | 1.459 (13) | Cl(2)-Ru(1)-N(2) | 93.2(2) |
| N(4)-C(12) | 1.362 (13) | Cl(3)-Ru(1)-N(2) | 91.0(2) |
| N(4)-C(17) | 1.463 (13) | Cl(4)-Ru(1)-N(2) | 87.0(2) |
| | | N(1)-Ru(1)-N(2) | 179.8(4) |
| | | Ru(1)-N(1)-N(3) | 130.1(6) |
| | | Ru(1)-N(1)-C(13) | 122.3(7) |
| | | N(3)-N(1)-C(13) | 107.4(7) |
| | | Ru(1)-N(2)-N(4) | 130.0(6) |
| | | Ru(1)-N(2)-C(14) | 122.9(7) |

[a]Numbers in parentheses are estimated standard deviations (Esd's) in the least significant digit

The N-Ru-N angle in **6** amounts to 179.8°. In **7**, the bond angle is about 174°, a slight reduction in comparison to **6**. The C atoms C(1) and C(1a) are shifted towards the center, and an optimal sterical positioning respective of the methyl groups on the opposite side is the result. There is a slight reduction of the Cl(1a)-Ru(1)-Cl(1) and N(2a)-Ru(1)-N(2) axes towards the $H_2O$. Considering the orientation of the Cl-Ru-Cl angles, a similar development can be observed: In compound **6** the Cl(1)-Ru(1)-Cl(2) and Cl(3)-Ru(1)-Cl(4) angles are 87.6° and the angles Cl(2)-Ru(1)-Cl(3) and Cl(1)-Ru(1)-Cl(4) are both 92.4°. In contrast to this, the angles in **7**, which surround the $H_2O$, are reduced to 86.3° (Cl(1a)-Ru(1)-O(1)), (Cl(1)-Ru(1)-O(1)). The angles on the opposite side are both still 93.7° ((Cl(2)-Ru(1)-Cl(1)) and (Cl(2)-Ru(1)-Cl(1a)).

**Table 5.** Selected Bond Distances (Å) and Angles (deg) of complex **7**; Not including the disordered cation.

| Bonds | Length[a] | Bonds | Angles[a] |
|---|---|---|---|
| Ru(1)-Cl(1) | 2.356 (4) | Cl(1)-Ru(1)-Cl(2) | 93.7(1) |
| Ru(1)-Cl(2) | 2.304 (6) | Cl(1)-Ru(1)-O(1) | 86.3(1) |
| Ru(1)-O(1) | 2.109 (17) | Cl(2)-Ru(1)-O(1) | 180.0(1) |
| Ru(1)-N(2) | 2.107 (8) | Cl(1)-Ru(1)-N(2) | 90.9(3) |
| Ru(1)-Cl(1A) | 2.356 (4) | Cl(2)-Ru(1)-N(2) | 92.8(3) |
| Ru(1)-N(2A) | 2.107 (8) | O(1)-Ru(1)-N(2) | 87.2(3) |
| N(1)-N(2) | 1.393 (13) | Cl(1)-Ru(1)-Cl(1A) | 172.6(2) |
| N(1)-C(7) | 1.382 (14) | Cl(2)-Ru(1)-Cl(1A) | 93.7(1) |
| N(1)-C(8) | 1.424 (17) | O(1)-Ru(1)-Cl(1A) | 86.3(1) |
| N(2)-C(1) | 1.315 (15) | N(2)-Ru(1)-Cl(1A) | 88.8(3) |
| | | Cl(1)-Ru(1)-N(2A) | 88.8(3) |
| | | Cl(2)-Ru(1)-N(2A) | 92.9(3) |
| | | O(1)-Ru(1)-N(2A) | 87.2(3) |
| | | N(2)-Ru(1)-N(2A) | 174.3(6) |
| | | Cl(1A)-Ru(1)-N(2A) | 90.9(3) |
| | | Ru(1)-N(2)-N(1) | 127.0(7) |
| | | Ru(1)-N(2)-C(1) | 123.2(8) |

[a]Numbers in parentheses are estimated standard deviations (Esd's) in the least significant digit

The Ru(1)-O(1) bond in **7** is shorter (2.109 Å) than the Ru-Cl bonds, which in both compounds are between 2.355 and 2.400 Å. The inclusion of a water molecule has no influence on the Ru-N bond lengths, which are on average 2.107 Å and, taking the Esd's into account, absolutely identical. This shows that in accordance with our investigations into the solution chemistry of compound **6** the formation of an aqua complex in a first hydrolysis step does not reduce the stability of the organic ligands and the substitution of another $Cl^-$ leaving group is likely to occur to give the bis-aqua complex.

**Biological tests.** After the successful synthesis of the indazole complex **1** with good yields and the necessary high purity, the positive results in biological test systems could be confirmed. Fig. 9 shows the results of the therapy of autochthonous colorectal tumors with complex **1** along with cisplatin, 5-fluorouracil and **ICR**, which has been the most active ruthenium species until now. The antitumor activity could even be surpassed with the indazole complex **1**, which effected a tumor reduction up to 90%. The 1-methyl complex **6** achieved similar T/C-values as **1** in the P388 leukemia. In this test system, complex **3** recently reached a good T/C-value of 160 % using a single dose of 75 mg/kg (0.116 mmol/kg) on three subsequent days after tumor transplantation.

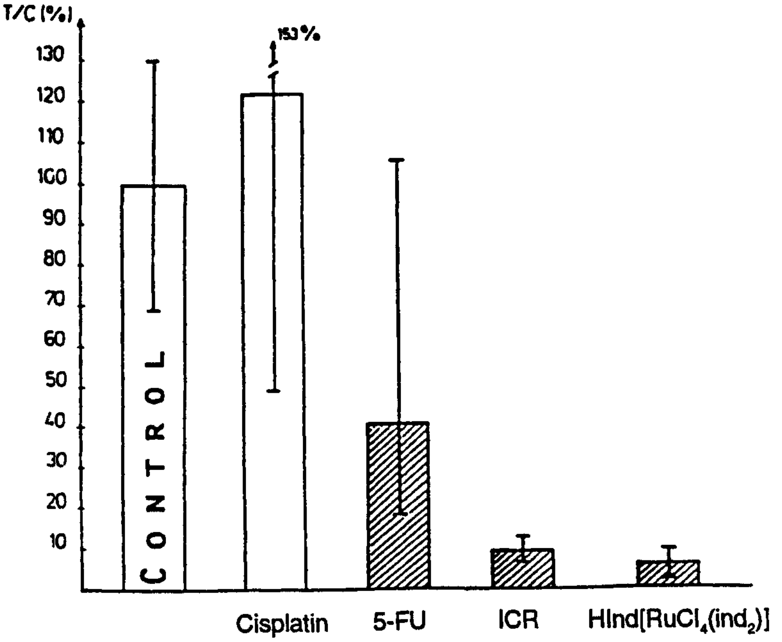

| | | | | |
|---|---|---|---|---|
| Dose (mg/kg) | 1.5 | 40 | 7 | 13 |
| mmol/kg | 0.0049 | 0.066 | 0.015 | 0.022 |

**Figure 9.** Test results of **ICR** and *trans*-HInd[$RuCl_4$(ind)$_2$] **1** in autochthonous colorectal tumors of the rat, compared to cisplatin and 5-fluorouracil. Doses were applied twice a week over ten weeks. The reduction of tumor volume represented by the shaded columns is statistically significant compared to the control group.

**Concluding Remarks.** The galenic behaviour of water-soluble ruthenium complexes is easier to manage, because adjuvants are not necessary in parenteral clinical applications. Furthermore the pseudo-first-order rate constants under physiological conditions show that these ruthenium compounds are sufficiently stable for intravenous infusion. This is a considerable advantage for later administration in the clinic. Another point is that freshly prepared solutions of **ICR** do not inhibit DNA polymerisation, whereas aged solutions, which had enough time to form aqua complexes, are able to inhibit DNA polymerisation to a high extent[22]. This makes it likely that many of the ruthenium compounds are only "prodrugs" and hydrolysis inside the tumor cell is necessary for activation.

As part of our program to synthesize water-soluble tumor-inhibiting ruthenium complexes, we have developed a synthesis for **1** with sufficient yield and high purity. The complex shows higher antitumor activity in all test systems than **ICR**, which has been the most active compound so far. The aquation of the anticancer complexes **ICR**[19,20] and **1** were studied extensively by means of UV-, IR- and NMR-spectroscopy as well as HPLC- and conductivity measurements. The data obtained shows that these compounds undergo aquation in aqueous solution, forming different hydrolysis products. For **ICR** we observed the formation of a neutral complex [$RuCl_3$(im)$_3$] as well as mono-aqua- and diaqua adducts. Due to the formation of the trisimidazole complex, the [$RuCl_4$(im)$_2$]$^-$

anion decreases continuously throughout hydrolysis. In contrast, complex **1** shows no tendency towards forming a trisindazole complex. The halflife of **1** was calculated to be 74.3 hours.
In the case of aquation of active complex **6**, we could resolve the resulting monoaqua species **7** crystallographically as well as the initial complex **6**. For the $RuCl_4L_2$-type prodrugs, this is the first time that a hydolysis product could be isolated to enrich the discussion of the relationship between the mode of action and structure. In case preclinical antitumor activity in colorectal tumors can be confirmed in the clinic, this would mean a considerable progress for the chemotherapy of cancer.

**Acknowledgements.** This work was supported by the Deutsche Krebshilfe, Dr. Mildred-Scheel-Stiftung für Krebsforschung, Bonn, Germany. We would like to thank Dr. Bernhard Nuber, Anorganisch-Chemisches Institut der Universität Heidelberg, for the X-ray crystal structure analyses. The excellent technical assistance of Martina Schickedanz is gratefully acknowledged.

### References

(1) M.J. Clarke, in: *Metal Complexes in Cancer Chemotherapy*, Ed. B.K. Keppler, VCH Weinheim, **1993**, 129.
(2) G. Mestroni, E. Alessio, G. Sava, S. Pacor and M. Coluccia, in: *Metal Complexes in Cancer Chemotherapy*, Ed. B.K. Keppler, VCH Weinheim, **1993**, 157.
(3) B.K. Keppler, K.-G. Lipponer, B. Stenzel and F. Kratz, in: *Metal Complexes in Cancer Chemotherapy*, Ed. B.K. Keppler, VCH Weinheim, **1993**, 187.
(4) E. Alessio, G. Balducci, A. Lutman, G. Mestroni, M. Calligaris and W.M. Attia, *Inorg. Chim. Acta* **1993**, *203*, 205.
(5) G. Mestroni, E. Alessio, G. Sava, S. Pacor, M. Coluccia and A. Boccarelli, *Metal-Based Drugs* **1994**, Vol. 1, No. 1, 43.
(6) F. Kratz, B.K. Keppler, L. Messori, C. Smith and E.N. Baker, *Metal-Based Drugs* **1994**, Vol. 1, No. 2-3, 169.
(7) R. Vilaplana, M.A. Romero, M. Quiros, J.M. Salas and F. Gonzalez-Vilchez, *Metal-Based Drugs* **1995**, Vol. 2, No. 4, 211.
(8) F. Kratz, B.K. Keppler, M. Hartmann, L. Messori and M.R. Berger, *Metal-Based Drugs* **1996**, Vol. 3, No. 1, 15.
(9) B.K. Keppler and W. Rupp, *J. Cancer Res. Clin. Oncol.* **1986**, *111*, 166.
(10) B.K. Keppler, D. Wehe, H. Endres and W. Rupp, *Inorg. Chem.* **1987**, *26*, 844.
(11) B.K. Keppler, W. Balzer and V. Seifried, *Arzneim.-Forsch./Drug. Res.* **1987**, *37*, 770.
(12) B.K. Keppler, W. Rupp, U.M. Juhl, H. Endres, R. Niebl and W. Balzer, *Inorg. Chem.* **1987**, *26*, 4366.
(13) F.T. Garzon, M.R. Berger, B.K. Keppler and D. Schmähl, *Cancer Chemother. Pharm.* **1987**, *19*, 347.
(14) B.K. Keppler, M. Henn, U.M. Juhl, M.R. Berger, R. Niebl and F.E. Wagner, *Progr. Clin. Biochem. Med.* **1989**, *10*, 41.
(15) J.M. Bakke and K.A. Skjervold, *Acta Chemica Scandinavia* **1975**, *B29*, 1089.
(16) DIF4, version 6.1., Meßprogramm für Siemens-Stoe AEDII-Vierkreisdiffraktometer, *Darmstadt, FRG*, **1984**.
(17) G.M. Sheldrick, SHELXTL-PLUS, *University of Göttingen, FRG*, **1988**.
(18) International Tables for X-Ray Crystallography, *The Kynoch Press, Birmingham* **1974**, *Vol. IV*.
(19) O.M. Ni Dhubhghaill, W.R. Hagen, B.K. Keppler, K.-G. Lipponer and P.J. Sadler, *J. Chem. Soc. Dalton Trans.* **1994,** 3305.
(20) J. Chatlas, R. van Eldik and B.K. Keppler, *Inorg. Chim. Acta* **1995**, *233*, 59.

(21) M. Henn, Dissertation, *Anorg.-Chem. Inst. Univ. Heidelberg, FRG*, **1989**.
(22) E. Holler, W. Schaller and B.K. Keppler, *Arzneim.-Forsch./Drug Res.* **1991**, 41(II), *10*, 1065.

**Received: September 22, 1996 - Accepted: September 24, 1996 - Received in revised camera-ready format: October 3, 1996**

